# Test and treat approach for tuberculosis infection amongst household contacts of drug-susceptible pulmonary tuberculosis, Mumbai, India

**DOI:** 10.3389/ftubr.2024.1454277

**Published:** 2024-09-18

**Authors:** Daksha Shah, Sampada Bhide, Rajesh Deshmukh, Jonathan P. Smith, Satish Kaiplyawar, Varsha Puri, Vijay Yeldandi, Anand Date, Melissa Nyendak, Christine S. Ho, Patrick K. Moonan

**Affiliations:** ^1^Brihanmumbai Municipal Corporation, Mumbai, India; ^2^TB Department, Society for Health Allied Research and Education (SHARE) India, Hyderabad, India; ^3^Division of Global HIV and TB, Centers for Disease Control and Prevention, New Delhi, India; ^4^Division of Global HIV and TB, Centers for Disease Control and Prevention, Atlanta, GA, United States

**Keywords:** tuberculosis, contact tracing, TB infection, interferon-gamma release assay, latent

## Abstract

**Background:**

Mumbai is one of the most densely populated areas in the world and is a major contributor to the tuberculosis (TB) epidemic in India. A test and treat approach for TB infection (TBI) amongst household contacts (HHC) is part of the national policy for TB preventive treatment (TPT). However, in practice, the use of interferon-gamma release assay (IGRA) tests for infection are limited, and prevalence of TBI in Mumbai is not known.

**Methods:**

We conducted a cross-sectional study among HHCs exposed to persons with microbiologically-confirmed, drug-susceptible pulmonary TB that were notified for antituberculosis treatment in Mumbai, India during September–December, 2021. Community-based field workers made home visits and offered IGRA (QuantiFERON-TB^®^ Gold In-Tube Plus) tests to HHC aged 5 years and older. After ruling out active TB disease, HHC with IGRA-positive test results were referred for TPT. All HHC were monitored for at least 24 months for progression to active TB disease.

**Results:**

Among 502 HHCs tested, 273 (54%) had IGRA-positive results. A total of 254 (93%) were classified as TBI and were eligible for TPT, of which 215 (85%) initiated TPT, and 194 (90%) completed TPT successfully. There was substantial variation in rates of TBI per household. In 32% of households, all HHC (100%) were IGRA positive and in 64% of households >50% of HHC were infected. In all, 22 HHCs (4%; 22/558) were diagnosed with TB disease; of these, five HHC were diagnosed during follow up, of which three were IGRA positive and had no evidence of disease at initial screening but chose not to initiate TPT.

**Conclusion:**

A test and treat strategy for HHC resulted in the detection of a substantial proportion of TBI and secondary TB cases. Home-based IGRA testing led to high participation rates, clinical evaluations, TPT initiation, and early diagnoses of additional secondary cases. A community-focused, test and treat approach was feasible in this population and could be considered for broader implementation.

## 1 Introduction

Tuberculosis (TB) remains a global public health crisis as one of the leading causes of death and disability worldwide (1). In 2022, World Health Organization (WHO) estimated that 10.6 million people suffered from TB disease, and 1.6 million died as a result (2). In addition to TB disease, approximately one in four individuals worldwide are presumed to have TB infection (TBI) (3). TBI is distinguished from active TB disease in that persons with infection are generally asymptomatic, lack clinical and microbial evidence of TB disease, and are unable to transmit *M. tuberculosis* bacilli to others (4). However, persons with infection harbor persistent bacilli and are at risk of progressing to TB disease. Approximately 5–10 percent of individuals with untreated infection will progress to TB disease over the course of their lifetime; thus, they are an important reservoir for future TB cases and continue to complicate global efforts to reduce TB incidence (3–7).

In high-burden countries, focusing on TB disease has historically been the priority of national efforts to reduce TB incidence. However, WHO's End TB Strategy—with ambitious goals to reduce global TB incidence by 90 percent by the year 2035—will not be possible without scaling up tuberculosis preventive treatment (TPT) efforts (8). When taken appropriately, TPT can reduce the progression from infection to active disease at a rate similar to the targeted progress needed to reach goal elimination (7, 8). TPT can substantially reduce ongoing transmission by eradicating *M. tuberculosis* before the progression to infectious forms of disease, and is generally shorter, less toxic, and more cost effective than treatment for disease (9). As a result, WHO has prioritized a comprehensive approach for the detection and treatment of TBI as an integrated approach to global TB control (5).

In 2022, India recorded the highest number of persons with TB and TB-attributed deaths in the world, accounting for almost a third of all cases (28%) and deaths (31%) globally (2, 10). An estimated 354 million people living in India are presumed to have TBI, and are at risk of becoming the next generation of future TB cases (3, 7). Mumbai is one of the most densely populated areas in the world and is a major contributor to the TB epidemic in India (11, 12). In 2022, the TB incidence rate in Mumbai was an estimated 313 per 100,000 population and accounted for ~105.5 notified TB cases per square kilometer as compared to 0.7 notified TB cases per square kilometer for all of India (10). As a consequence, identifying and treating individuals with TBI, especially household contacts (HHC), is a core component of India's National Strategic Plan (2017–2025) and the TB-Free Mumbai Plan (12, 13). This approach of “*test and treat*” is part of the national policy for TPT in India (14). Unfortunately, in practice the availability and use of high-quality tests for infection are limited, but the absence of these tests does not preclude or defer offering TPT to HHC (14). Moreover, the prevalence of TBI in Mumbai is not known.

We identified HHC of persons with microbiologically-confirmed pulmonary TB (PTB) in Mumbai, India and tested those aged 5 years and older for TBI. The primary goal of this study was to estimate the prevalence of TBI among these HHC, as well as to determine the feasibility of implementing tests for infection (i.e., interferon-gamma [IFN-γ] release assay [IGRA]) in a programmatic setting. IGRA-based testing is preferred in countries where BCG vaccination at birth is national policy, as test results are not confounded by BCG (5).

## 2 Methods

### 2.1 Study design, setting, and population

We conducted a cross-sectional study among HHCs exposed to persons with microbiologically-confirmed PTB that registered for antituberculosis treatment (ATT) in Mumbai, India during September–December, 2021. During the study period, we randomly selected persons with drug-susceptible PTB tested by cartridge-based nucleic acid amplification test (CBNAAT; Molbio Truenat MTB, Truenat MTB Plus, or Truenat Rif-Dx, Goa, India) from each of the 24 wards in Mumbai. We randomly selected index patients based on a ward-specific quota to ensure a geographically representative sample of households in Mumbai, and we capped enrolment to reach sufficient power to measure TBI prevalence of HHC. The HHC sample size was derived from a national average household size in India of 4.8 persons (15). Estimates assumed a large (0.50) intraclass correlation (ICC) between household members, and α and β levels of 0.05 and 0.20, respectively. Sample size estimates were produced with binomial confidence limits applying a design effect (1+[μ-1]^*^ICC), where μ was the average number of members per cluster (here, contacts per household), and ICC was the intraclass correlation coefficient measuring the correlation among members of the same household. We estimated needing to sample at least 125 microbiologically positive TB index patients assuming TBI prevalence estimates of 40–70%, varying the average number of contacts per household and the total number of households needed to yield a minimum of 500 contacts. All HHC aged 5 years and younger were evaluated and treated with TPT without a test for infection and not included in this analysis.

We defined an index patient as an individual with microbially-confirmed, drug-susceptible PTB registered for treatment at the Brihanmumbai Municipal Corporation (BMC) TB Program, the local health authority for Mumbai. We defined HHC as a person who shared the same living space with an index patient for at least one night or had daytime periods of exposure in the home that occurred during the 3 months before the start of ATT of the putative index patient.

### 2.2 Home visits, contact elicitation, and IGRA testing

Trained field coordinators identified, counseled, consented, and enrolled HHCs of persons with microbiologically confirmed drug-sensitive pulmonary TB during home visits. As per national guidelines, IGRA tests (QuantiFERON-TB^®^ Gold In-Tube Plus, [QFT-GIT Plus], QIAGEN, Hilden, Germany) were offered to all HHC aged 5 years or older (14). During home visits, and after consenting, trained phlebotomists collected ~3 ml of venous blood directly into QFT-GIT Plus vials. Blood was transported to King Edward Memorial Hospital laboratory within 12 h of collection and incubated for 16 to 24 h at 37°C prior to harvesting stimulated plasma for ELISA processing. The concentration of IFN-γ in 50 μl of each plasma sample was determined as per the manufacture's manual of procedures and international guidelines (16). IGRA test results were interpreted as positive when the IFN-γ concentration was ≥0.35 IU/mL and ≥25% of the nil value (16). A standardized four symptom screening tool was utilized to assess HHC for clinical signs and symptoms consistent with TB disease (17).

Symptomatic HHCs or those with positive IGRA results were referred for chest radiography (CXR). Persons with symptoms or abnormal CXR were presumed to have TB and referred for additional evaluation and subsequent initiation of ATT, as per the National TB Elimination Program (NTEP) guidelines (17). After ruling out active TB disease, HHC with positive IGRA test results were referred for TPT (once weekly isoniazid and rifapentine for 12 weeks [3HP] or daily isoniazid for six months [6H]), as per NTEP guidelines (14). All CXRs, IGRA tests, and clinical services were offered at no additional cost. However, CXR and clinic visits may have required local travel and time off from work.

### 2.3 Follow-up evaluations, frequency, and periodicity

Study field coordinators supported BMC NTEP staff to conduct semi-annual home visits for up to 2 years (1 September 2021–31 December 2023) to support ATT or TPT and to monitor HHC for the progression to TB disease. HHCs with symptoms consistent with TB during follow-up were further evaluated for disease and managed as per NTEP guidelines (17).

### 2.4 Statistical analysis

Frequencies and proportions were used to describe categorical variables; medians and interquartile ranges (IQR) were used to describe continuous variables. We examined bivariate associations of HHC demographic and clinical characteristics, index patient characteristics (including treatment delays exceeding 1 week and whether the TB episode was new or a retreatment), and household characteristics (including whether the household income fell below the poverty line,^*^ located in a slum,^†^ and the presence of additional persons with concurrent TB) and positive IGRA test results. We used mixed-effects logistic regression with a random effect for household to calculate odds ratios (OR) and 95% confidence intervals (95% CI) to measure associations of interest; values that did not cross the null (1.0) were consider statistically significant.

### 2.5 Ethical approval

This study was reviewed and approved by BMC local ethics committee, SHARE India-Mediciti ethics committee. Written informed consent was obtained by participants or their legal guardians.

## 3 Results

### 3.1 Index patient selection and household contact elicitation

**Figure 1:**
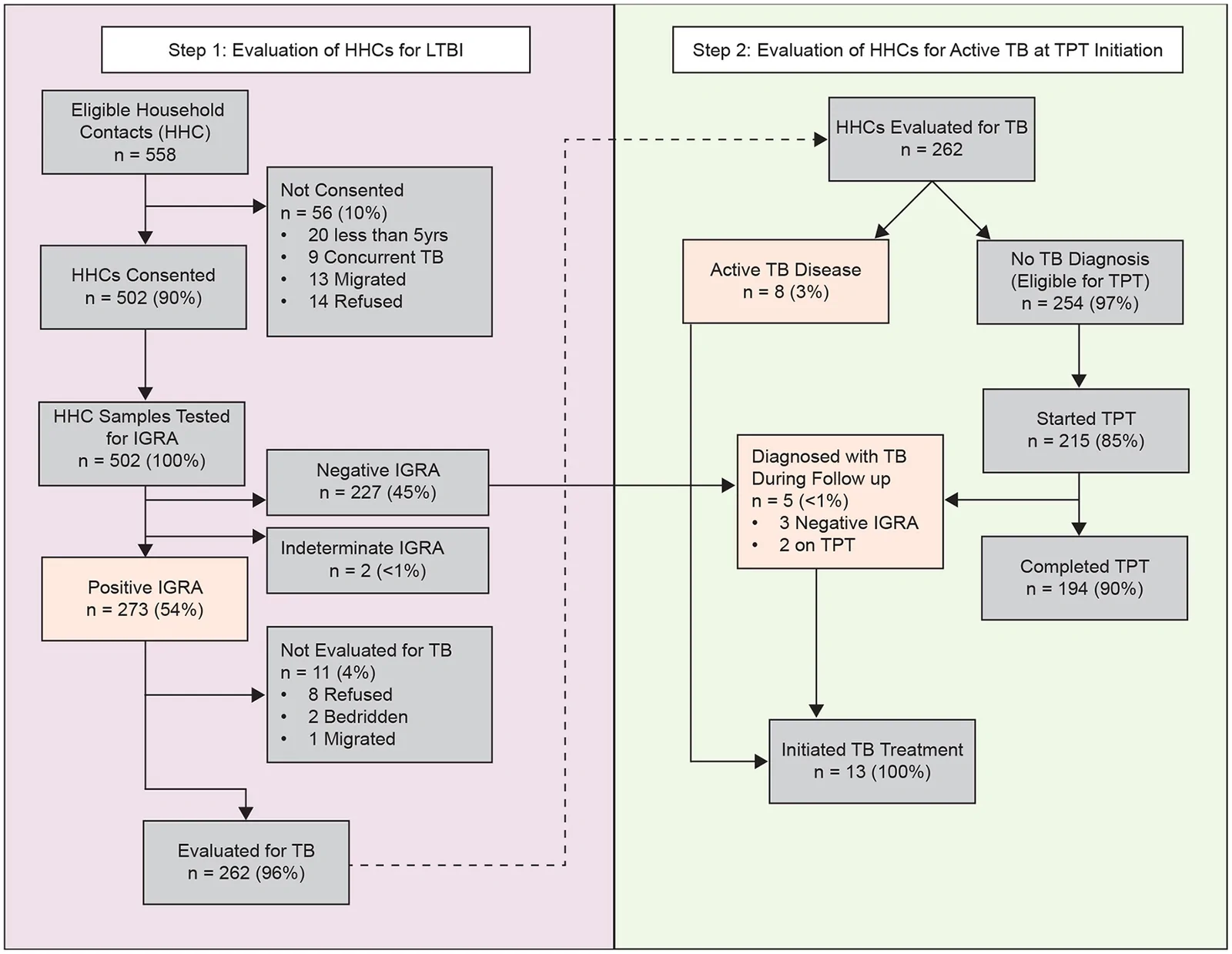
Identification, enrolment, evaluation, follow-up outcomes of household contacts of pulmonary tuberculosis, Mumbai, India—September 2021–31 December, 2023. Participants evaluated for active TB disease included those with positive IGRA results and received a chest radiograph. Highlighted boxes (orange) indicate primary outcomes (TB infection or active TB disease). HHC, household contact; IGRA, interferon-gamma release assay; TPT, tuberculosis preventive treatment.

One hundred and fifty-eight index patients were selected and enrolled during the study period (Supplementary Table). A total of 558 HHC were identified, 20 (3.6%) were < 5 years of age, 14 (2.5%) refused enrolment, 13 migrated away from Mumbai, and nine (1.6%) had co-prevalent TB at baseline (Figure 1). Thus, 502 HHC (90%) were deemed eligible for IGRA testing. The median household size was 4 persons (IQR: 3, 5); 92% of households were located in a slum. Among index patients, the median age was 35 years (IQR: 21, 47) and 15 (9%) had multiple episodes of TB disease. The majority of both index patients and HHCs were women (59% and 51%, respectively).

### 3.2 Tuberculosis infection and case detection at baseline

**Figure 2:**
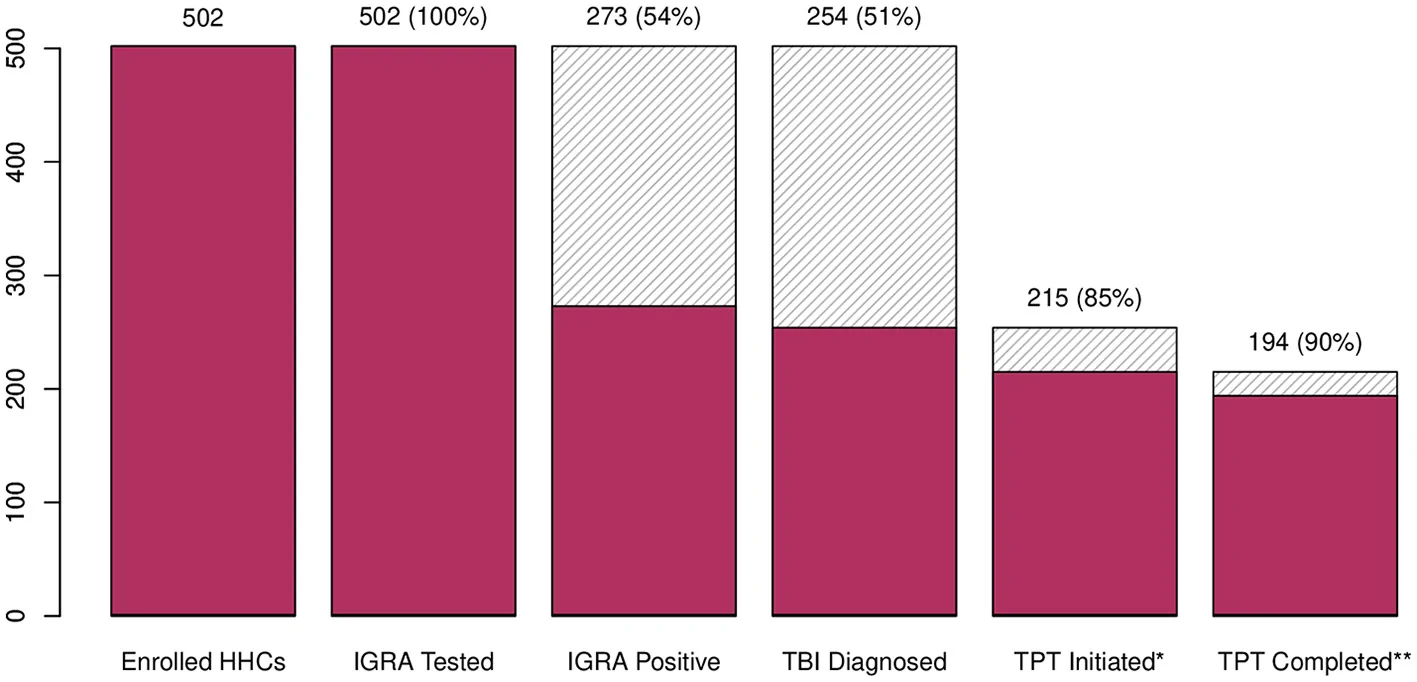
TB infection and treatment cascade—Mumbai, India, 2021–2023. HHC, household contact; IGRA, interferon-gamma release assay; TBI, tuberculosis infection; TPT, tuberculosis preventive treatment; 3HP, once weekly isoniazid and rifapentine for 12 weeks; 6H, daily isoniazid for 6 months. *177 HHC initiated 3HP regimen; 38 HHC initiated 6H. **14 (8%) HHC on 3HP regimen prematurely discontinued: five had an adverse drug events (i.e., headache, fatigue, skin rash, vomiting), five refused to continue, two transferred out of Mumbai, and two developed pulmonary tuberculosis during follow-up; 7 (18%) HHC on 6H regimen prematurely discontinued: one had an adverse drug events (i.e., fever, skin rash), and six refused to continue.

Among the 502 HHCs tested, 500 (99%) had interpretable IGRA results, of which 273 (54%) had immunological evidence of TBI (Figure 1). Among these, 262 (96%) were initially evaluated by CXR; eight (3%) had radiologic evidence of pulmonary TB, and 254 (97%) had no evidence of pulmonary TB, classified as tuberculosis infection, and were eligible for TPT. Among those with TBI, 215 (85%) initiated TPT, 33 (13%) refused, six (2%) relocated away from the jurisdiction, and one was pregnant and chose not to initiate TPT. In total, 194 of the 215 (90%) HHC who initiated TPT completed the full course (Figure 2). The median time from index case diagnosis to treatment initiation was 2 days (IQR: 1, 4 days). The median time from HHC elicitation to IGRA testing was 3 days (IQR: 2, 4 days) and 14 days (IQR: 8, 23) from IGRA testing to TPT initiation.

### 3.3 Progression to active disease during follow-up

**Figure 3:**
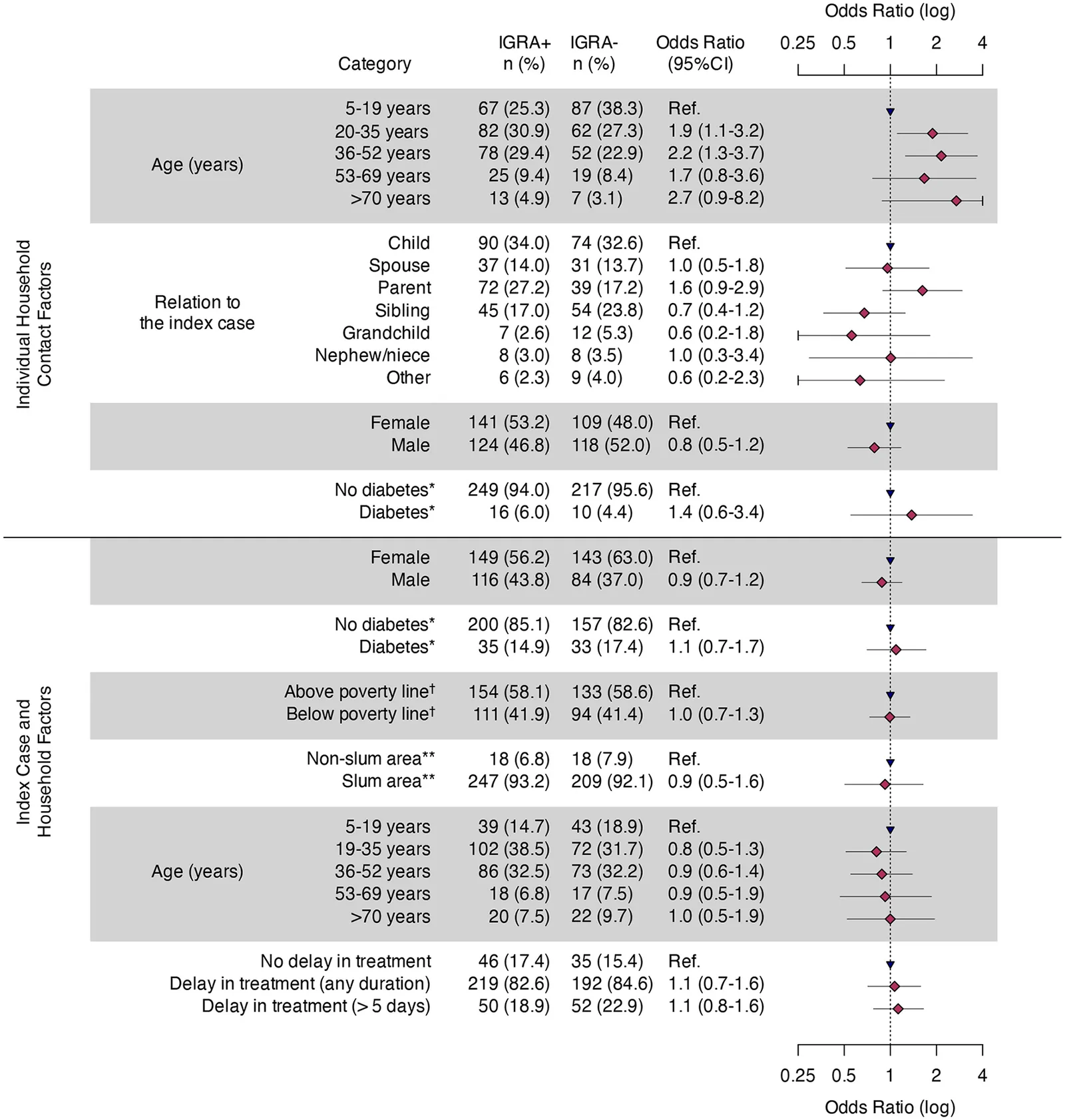
Odds of tuberculosis infection stratified by individual, index patient and household characteristics—Mumbai, India, 2021–2023. HHC, household contact; TBI, tuberculosis infection. *Of the 158 index cases, 25 were missing diabetes status, accounting for a total of 67 HHCs. Thus, the final HHC count for this covariate is 425 (all others are 492). ^†^Poverty line = <?32 per day. **Slum defined as a compact settlement of at least 20 households with a collection of poorly built tenements, mostly of temporary nature, crowded together usually with inadequate sanitary and drinking water facilities in unhygienic conditions. https://mohua.gov.in/upload/uploadfiles/files/9Slum_Report_NBO(2).pdf.

**Figure 4:**
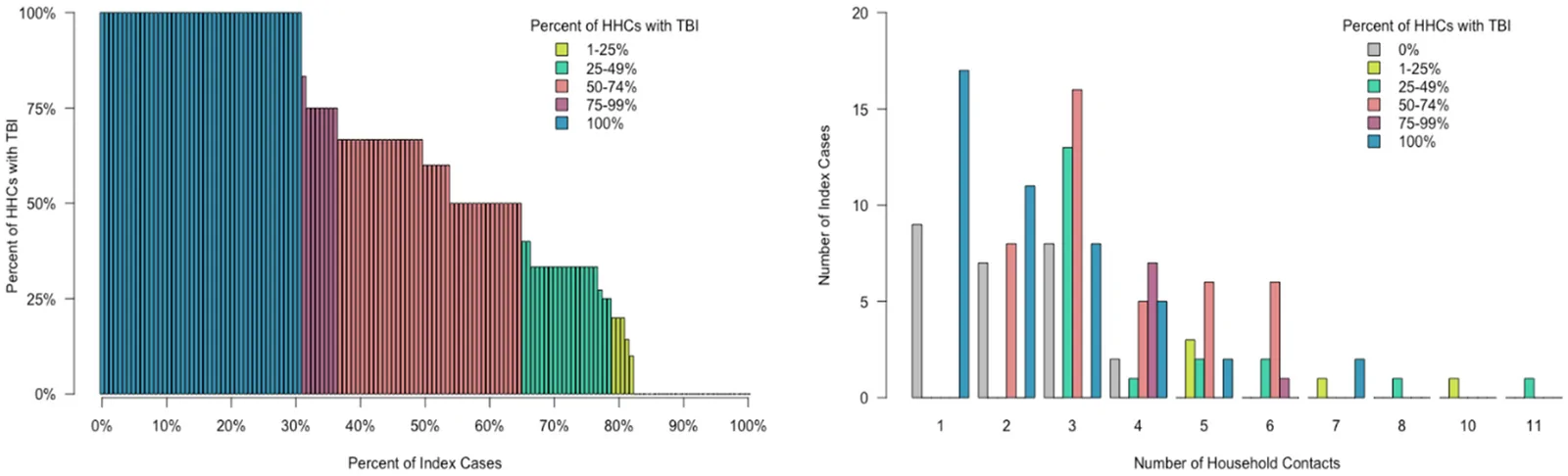
Tuberculosis infection rates by household size—Mumbai, India, 2021–2023.

During follow up, five HHC progressed to TB disease. Among the five HHC diagnosed with TB disease during follow up, three HHC (60%) were IGRA-negative and had no evidence of disease during the initial screening (one did not receive CXR due to pregnancy). The remaining two were IGRA positive and initiated TPT but progressed to disease during 6 and 12 months of follow up, respectively. Thus, a total of 22 (4%) HHCs (i.e., nine had co-prevalent TB at baseline, eight had concurrent TB disease at initial evaluation, and five progressed to TB disease during follow-up) were diagnosed with TB disease (Figure 1). All had drug-susceptible pulmonary TB, and were initiated multi-drug, multi-month ATT as per national guidelines (17). We found no statistical difference in clinical (of the index patients), or household factors associated with the number of HHCs with TBI (Figure 3). However, older HHC (aged 19–52 years) had greater odds of TBI than younger HCC (aged 6–19 years). There was substantial variation in the proportion of TBI per household (Figure 4). In 32% of households all HHC (100%) were IGRA positive, and in 64% of households more than half of HHC were infected. Sixteen percent of households had no (0%) HHC with measurable infection — interestingly, this occurred only in households with four or fewer contacts.

## 4 Discussion

India, a high-priority country for achieving the United Nation's Sustainable Development Goals, has committed to ending TB (18, 19). Expanding testing and treatment of tuberculosis infection is critical to achieving this goal (7, 8, 10, 12, 13). In this high-burden, high population density setting, we found the majority of HHC (54%) tested positive for TBI using IGRA. Among these, almost all (96%) were further evaluated by CXR, and eight had concurrent TB disease at baseline. The vast majority of HHC with IGRA-positive results both initiated and successfully completed a full course of TPT.

The prevalence of TBI in our cohort, as measured by IGRA-based testing, was greater than other similarly designed studies of HHC in India. Project Axshya (ten project districts across Maharashtra and Himachal Pradesh) found IGRA positivity in 26% of exposed HHC (20). A nested IGRA-based analysis within the recent national TB prevalence study found similar proportions of IGRA-positive results amongst HHC (27%) and non-HHC (24%) (21). In these studies, the prevalence of TBI among HHCs were similar to the prevalence of the general population of India [pooled prevalence: 36% (95% CI: 28–45%)] (22) and the Asian region [pooled prevalence: 21% (95% CI: 19–23%)] (23). In both of the India-based HHC studies, approximately, one in five persons eligible for IGRA-based testing, refused or did not present for phlebotomy. We hypothesize that our superior testing acceptance rate (99%) might be attributed to the home-based nature of our services, but it is unclear if this influenced our relatively higher prevalence estimates. What is clear, is the transition from passive screening and testing that places responsibility on the HHC to present for care to actively screening and testing at places and times convenient to them has improved outcomes elsewhere (24). In a similar study from Indonesia, the TBI prevalence amongst HHC was slightly higher (59%) and more closely reflects the prevalence of this high-priority group in a high-burden setting (25). It is intuitive that the prevalence of TBI in HHC should be greater than the general population (22, 25–27). This may be even more important in high-population density communities, like the slums of Mumbai, where the prevalence of other respiratory infections was greater than non-slum areas (28). In a densely-populated urban slum along the U.S.-Mexico border, 57% of at-risk enrollees had positive IGRA test results (29), but substantially lower rates [39%] were found in non-slum areas of the same community (30). It is also intuitive as the individual and community force of infection increases so does the risk of positivity (31). Prioritizing targeted test and treat campaigns among households with more than one pulmonary case might be an effective first step to reduce TB transmission for programs with limited resources or capacity, especially in high-burden neighborhoods (32). Moreover, *M. tuberculosis*-specific, antigen-based skin tests, such as Cy-TB (Serum Institute of India, Pune, India), could increase access to tests for TBI with high specificity and lower infrastructure and laboratory costs (33).

TPT initiation (85%) and completion (90%) rates among our cohort were also higher than experienced in most previous Indian studies (34–39). This improvement might be due to the availability of short-course TPT regimens, expanding TPT eligibility to include adults, and the frequent interactions associated with our person-centered, home-based monitoring, which demonstrated similar success elsewhere (40). Community-based applications of short-course TPT in other high-burden communities that used self-administered options, experienced minimal side effects, and also achieved high completion rates (41–43).

It is important to note that more than a third (5 of 13) of all concurrent pulmonary TB cases, including three (60%) HHC that were initially IGRA negative, were discovered during follow-up monitoring. This reinforces that contact tracing is not a one-time event, but rather a series of multiple observations and evaluations post exposure (44). Test and treat programs should consider including resources for follow up testing between 8 and 10 weeks after the last exposure to the case (16, 44). If the result of the repeat test is positive, and after ruling out active disease, the HHC should be considered a recent infection and prioritized for TPT (16, 44). Of note, measuring immunologic response to *M. tuberculosis* exposure is believed to be transient, can lead to potential conversions and reversions, and does not always predict TBI or progression to disease (4, 45–48). Moreover, the exact incubation period of TB varies based on individual age, immunity (e.g., HIV), comorbid clinical conditions, treatments (e.g., diabetes, end-stage renal disease, immunosuppressive medications, silicosis, and undernutrition), and behaviors (e.g., excessive alcohol use, illicit substance use, and smoking) (4, 47–49). However, disease progression typically occurs within several months to two years, and after that, disease progression is relatively infrequent (50, 51). Long-term monitoring of HHC could be easily incorporated during the care and treatment of the household index patients (and potential secondary cases), and as part of post-treatment surveillance processes (13, 52).

Programs with adequate resources should consider expanding contact tracing beyond the home when infection rates are higher than expected following a concentric circle approach (53) or contact priority models (54) which expand test and treat to additional contacts based on the background community tuberculosis infection rates. Our finding that 64% of households had more than half of the contacts testing positive suggest high rates of transmission, and warrants expansion of testing to others to fully understand the scope of risk in the larger community. Additional tools are available to help expand contact tracing using quantifiable exposure variables based on person, place, and time, or relative infectiousness of the index patient (55–57).

This study had several limitations. First, our sampling methodology was non-probabilistic and there was potential for survey bias. Additional studies are needed to ascertain the prevalence of TBI in the general population of non-HHC in Mumbai. Second, an unknown proportion of asymptomatic persons could have been missed by the screening criteria and may have resulted in an underestimation of TB disease among HHC. Third, contact tracing is an imperfect public health intervention (58). It is likely, despite our best efforts to elicit contacts, conducting home visits, and offering free services, some important HHC might have failed to present for evaluation. These missed HHC might affect our estimates of TBI prevalence and potentially led to the underdiagnoses of related secondary cases (31, 59). Moreover, living with an index patient might not always translate into time spent together, and is not the only opportunity for TB exposure in a high-burden community. An index patient is the person for which a contact investigation is centered upon but is not necessarily the source patient (60). Thus, some of the TBI prevalence observed could be attributable to non-household exposure to other persons with TB disease. Lastly, field activities were funded through a cooperative agreement with an implementing partner that augmented testing and other TB services delivered by the local health department. These additional resources may not reflect the ground reality of government-funded TB services in Mumbai.

## 5 Conclusions

A test and treat strategy among HHC resulted in the detection of a substantial proportion of TBI and secondary TB cases. Home-based IGRA testing observed high participation rates, clinical evaluations, and TPT initiation. Frequent and regular monitoring likely aided TPT compliance, contributed to early diagnoses of additional secondary cases, and may have interrupted TB transmission in a high-burden community. A community-focused, test and treat approach was feasible in this population and could be considered for broader implementation.

## Data Availability

The raw data supporting the conclusions of this article will be made available by the authors, without undue reservation.

## Footnotes

^*^Below the poverty line was defined as earning less than ₹ 32 per day.

^†^Slum was defined as a compact settlement of at least 20 households with a collection of poorly built tenements, mostly of temporary nature, crowded together usually with inadequate sanitary and drinking water facilities in unhygienic conditions (11).
