# Supplementary material for: Test and treat approach for tuberculosis infection amongst household contacts of drug-susceptible pulmonary tuberculosis, Mumbai, India
Source: Front Tuberc. 2024 Sep 18;2:1454277. doi: 10.3389/ftubr.2024.1454277 (PMC11485165; doi:10.3389/ftubr.2024.1454277)
Supplement: Supplementary file 1 [file Table_1.pdf]

Supplemental Table. Number of index cases and household contacts enrolled by district — Mumbai, India, 2021–2023.

District Name (n=24)	Number of index cases (n=158)	Number of household contacts (n=502)	Average number of household contacts per index case
ANDHERI EAST	13	35	2.7
ANDHERI WEST	12	28	2.6
BAIL BAZAR ROAD	12	56	4.1
BANDRA EAST	5	9	2.3
BANDRA WEST	2	9	4.5
BORIVALI	5	12	2.6
BYCULLA	4	10	2.5
CENTENARY	6	24	4.0
CHEMBUR	11	21	3.0
COLABA	3	9	3.0
DADAR	5	19	3.6
DAHISAR	5	16	3.0
GHATKOPAR	7	22	3.1
GOREGAON	5	10	2.0
GOVANDI	10	49	3.6
GRANT ROAD	1	1	1.0
KANDIVALI	17	67	3.9
KURLA	6	16	3.8
MALAD	9	34	3.2
MULUND	1	3	3.0
PAREL	4	8	2.6
SION	6	16	2.7
VIKHROLI	9	28	3.1
PRABHADEVI*	0	0	0
**Grand Total**	**158**	**502**	**3.2**

*Among five eligible index cases, no household contacts were enrolled (two index cases refused enrolment, two transferred out before contact elicitation, and one lived alone).
